# Nursing care to patients who have the home as the preferred place of death: a scoping review

**DOI:** 10.1186/s12913-024-11757-8

**Published:** 2024-10-29

**Authors:** Anne Kristine Sørstrøm, Ingjerd Gåre Kymre, Mette Spliid Ludvigsen

**Affiliations:** 1 Faculty of Nursing and Health Sciences, Nord University, Bodø, Norway; 2 Nordland Hospital Trust, Bodø, Norway; 3 Department of Clinical Medicine - Randers Regional Hospital, Aarhus University, Aarhus, Denmark

**Keywords:** Patients who prefer to die at home, healthcare systems, nursing care, palliative care, home settings

## Abstract

**Background:**

The existing literature on nursing care for patients who choose home as their preferred place of death is scattered and lacks a coherent overview. This scoping review aimed to explore and categorize the available evidence on how nurses provide care for patients preferring to die at home.

**Methods:**

Studies that included nurses and were focused on nursing care for patients who choose the home as their preferred place of death were included in the review. The scoping review considered studies with quantitative, qualitative, or mixed method designs; systematic reviews; and meta-analyses. No time restrictions were added. Key information sources were Medline, CINAHL (EBSCO), Scopus (Elsevier) and Google Scholar. Systematic reviews were searched for in the Cochrane Database of Systematic Reviews. Unpublished studies and grey literature were searched for in ProQuest Dissertations and Theses. The reference list of the studies included was searched.

**Results:**

A total of 13 studies were deemed eligible for inclusion in the review, of which (*n* = 11) were qualitative and (*n* = 2) were both qualitative and quantitative. The studies were published between 2008 and 2023 and were conducted in the United Kingdom (*n* = 5), Norway (*n* = 4), Australia, Sweden, Canada and Japan. The studies included in this review highlighted issues of competence, resource limitations, flexibility as a coping mechanism, as well as collaboration and family caregivers.

**Conclusions:**

This review identified significant challenges in delivering nursing care for patients who prefer to die at home, including staff shortages, resource limitations, and educational deficiencies. Despite these barriers, nurses showed a strong commitment to patient care, highlighting the need for increased support and collaboration with family caregivers to improve home-based end-of-life care.

**Implications for research:**

To improve care for patients who wish to die at home, it is crucial to address staff shortages and enhance nurse training to close knowledge gaps and ensure consistent, high-quality care. Healthcare systems must also allocate adequate resources to ensure that nurses have the necessary tools to deliver safe and effective care in home settings. Strengthening interdisciplinary collaboration will further enhance patient outcomes by supporting both nurses and family caregivers in end-of-life care.

**Supplementary Information:**

The online version contains supplementary material available at 10.1186/s12913-024-11757-8.

## Introduction

Research shows that many patients want to be cared for in their own home at the end of life and to die at home [1–3]. While a death at home is not desirable or feasible for everyone, healthcare professionals can accommodate patients who want to be cared for at home in cases where this can be a good option [4]. A population-based study from 2015 identified a crucial factor for patients dying at home: the provision of community nursing during the last three months of life [5]. Expanding the availability of home care services is likely to give individuals a greater say in decisions related to their place of death, thereby enabling more people to die at home if that is their preference [6].

Due to increase in the elderly population, more people will be cared for in community home-based care and a growing number will die at home [7]. Hence, an increase in the number of people in need of palliative care at home can be expected. Projections indicate that if current trends persist, the demand for end-of-life care will increase significantly over the next 25 years, especially in home and care home settings [8]. As the demand for end-of-life care grows, it is becoming increasingly crucial to focus on the resilience, capacity, and training of the health and social care workforce, especially within community settings [8].

A systematic review found that patients want responsive palliative care services, organised to meet their preferences. This includes help that responds to their needs, as well as coordinated services that ensure continuity [4]. Nurses play a crucial role in caring for patients who wish to die at home. While the title of this role varies across countries—such as district nurse, community nurse, or home care nurse—these professionals consistently provide general nursing care in domestic settings, addressing a broad range of health and nursing needs, including end-of-life care and home deaths [9]. However, with the rise of contemporary care models, such as virtual wards, hospital outreach, and specialized teams for specific conditions, the traditional role of nurse in the home care service is evolving. This scoping review seeks to explore how nurses provide care for patients who prefer to die at home within this shifting landscape, capturing both conventional practices and emerging care models.

Many municipalities have limited resources, with few nurses in proportion to the workload, restricted night service, and nurses that often lack sufficient training in palliative care [10–12]. A review of palliative care in rural Canada and Australia reported stress factors among nurses such as geographical isolation, infrequent cases of palliative care patients, and blurred work boundaries [13]. According to a recent global survey, significant barriers to providing palliative home care are personnel shortages, lack of funding and policies, lack of time for education and diminished recognition in society of the palliative care provided [14]. An integrative literature review of the experiences of Australian nurses providing palliative care in rural settings for patients wanting to die at home reports a lack of insight into the complexity of the work of nurses providing home-based palliative care, and advises further exploration to sustain and improve palliative care for patients wanting to die at home [15].

Given the growing demand for high-quality palliative care, regardless of diagnosis, and the increasing focus on place of death, it was deemed important to map the available evidence on how nurses provide care for patients who prefer to die at home. This scoping review seek to enhance understanding of the complexities surrounding access to end-of-life care in home care services. By organizing fragmented literature and identifying diverse care practices, it will support evidence-based interventions, and perhaps inform policies.

Preliminary database searches, including the Joanna Briggs Institute (JBI) Database of Systematic Reviews, revealed no scoping reviews that systematically map studies on nursing care for patients preferring home as their place of death.

## Methods

The aim of this scoping review is to identify and systematize the existing evidence on nurse’s care for patients who have home as their preferred place of death. An a priori protocol was published to guide this review [16]. Scoping review methodology was considered suitable to systematically map the range of literature, to identify key concepts and identify the types of evidence available [17]. The review was conducted in accordance with the JBI methodology for scoping reviews and was reported according to the Preferred Reporting Items for Systematic reviews and Meta-Analyses extension for Scoping Reviews (PRISMA-ScR) checklist [18].

### Study criteria

#### Participants

In this review we included studies focusing on nurses caring for adult patients (aged 18 years or more) who have home as their preferred place of death, regardless of diagnosis., We considered for inclusion studies focusing on but not limited to home care nurses, district nurses, community nurses, visiting nurses and primary care nurses were considered for inclusion. For readability, we will refer to the participant role as “nurses” in this study. We excluded studies that focus on other healthcare professionals.

#### Concept

The home as the preferred place of death is in this review is understood as a “*planned*,* expected home death where an individual has chosen to die at home with the support of family and plans have been clearly made and documented beforehand*” [19]^,p2^. Numerous studies emphasise that the objective of home-based palliative care is not solely focused on achieving death at home, but rather on enabling patients to remain in the comfort of their homes for as long as they desire. Many articles pertain to home-based palliative care across various stages of the palliative care continuum; however, the concept of this scoping review is the nursing care for patients who prefer home as their place of death.

#### Context

This scoping review included studies in the context of home care services. Other contexts than in the home (such as nursing homes, long term care facilities or hospices) were excluded.

#### Types of sources

For this scoping review we considered studies utilising qualitative, quantitative and/or mixed methods design, systematic reviews and meta-analyses, PhD theses and dissertations, text and opinion papers, and unpublished studies. Conference papers were excluded. Studies published in the English language were included.

#### Information sources

Five databases that were considered relevant for this review were searched: MEDLINE (EBSCOhost)), CINAHL (EBSCOhost), Scopus (Elsevier) and Google Scholar. Systematic reviews were searched for in the Cochrane Database of Systematic Reviews and unpublished studies were searched for in ProQuest Dissertations and Theses. The reference list of included articles was searched to identify additional relevant studies.

#### Search strategy

We performed a preliminary search to identify relevant MeSH terms, keywords and synonyms, different terminology, word plurals, different wordforms and different word spellings, common acronyms, and common vs. specific names. A university librarian was consulted to identify relevant and up-to-date search words. We used PubMed to explore relevant subject headings. We used truncations where applicable. Systematic searches were performed from November to January 2019 and updated January 2024. The searches were evaluated and validated in consensus by the authors before running them in each of the databases. We searched for records in the English language, and search terms included MeSH terms and keywords, as appropriate, depending on the database searched. We did not use time restrictions. The last search was performed on 19 January 2024. The search strategy for MEDLINE is shown in Appendix I.

#### Study selection

Following the search, all identified records were uploaded into EndNoteX9 (Clarivate Analytics, PA, USA) and duplicates were removed. The citations were then uploaded in Rayyan systematic review software for screening [20]. Titles and abstracts of the records underwent independent screening by two reviewers, based on inclusion criteria. Reports for full text reading were acquired, comprehensively examined for eligibility, and included reports made subject to citation tracking to identify additional records meeting the inclusion criteria. Articles had to be reviewed in full text to confirm they specifically addressed nursing care for patients with home as their preferred place of death, rather than general palliative care at home. Due to the fluidity of terms related to palliative care, home death, and end-of-life care, it was challenging to distinguish between studies focused on dying at home and those on general home-based palliative care. As a result, many articles underwent full-text screening to accurately identify their relevance. This necessitated a broader inclusion approach in the initial screening phase to ensure that relevant studies were not overlooked. Any discrepancies in the selection process were resolved through discussion among all authors until a consensus was reached.

#### Data extraction

Data mapping was conducted by the first author (AKS), with support from co-authors (IGK, MSL). The review team pilot assessed the process to ensure the consistency of the approach, by comparing [21] extraction notes for a chosen study [17]. The data extracted enabled charting of both general information and information that best answered the aim of the review. A standardised data extraction form was developed and utilised to chart data for authors/year/country, aim, study population, setting, methodology and data collection (Table 1).

For the qualitative data, we employed qualitative content analysis, as suggested in JBI scoping review guidance [21, 22]. Using an inductive approach [23], we applied open coding to identify concepts and categorize them according to the research question. We chose an inductive approach, as this has been deemed beneficial when there is a dearth of evidence regarding the subject matter [24]. We then began familiarising ourselves with the data, by reading and comprehending the evidence sources included, and focusing on how they were relevant to the review questions. During the analysis, we identified commonalities and distinctions within the data, organising them into categories or themes at different levels of abstraction and interpretation. We transitioned from the specifics of the data to a broader theoretical comprehension, moving from the concrete and particular to the abstract and universal [23]. Analysis and summarisation of individual coding (sections designated with a specific code) were conducted, providing a narrative overview for each coded topic.

## Results

**Fig. 1:**
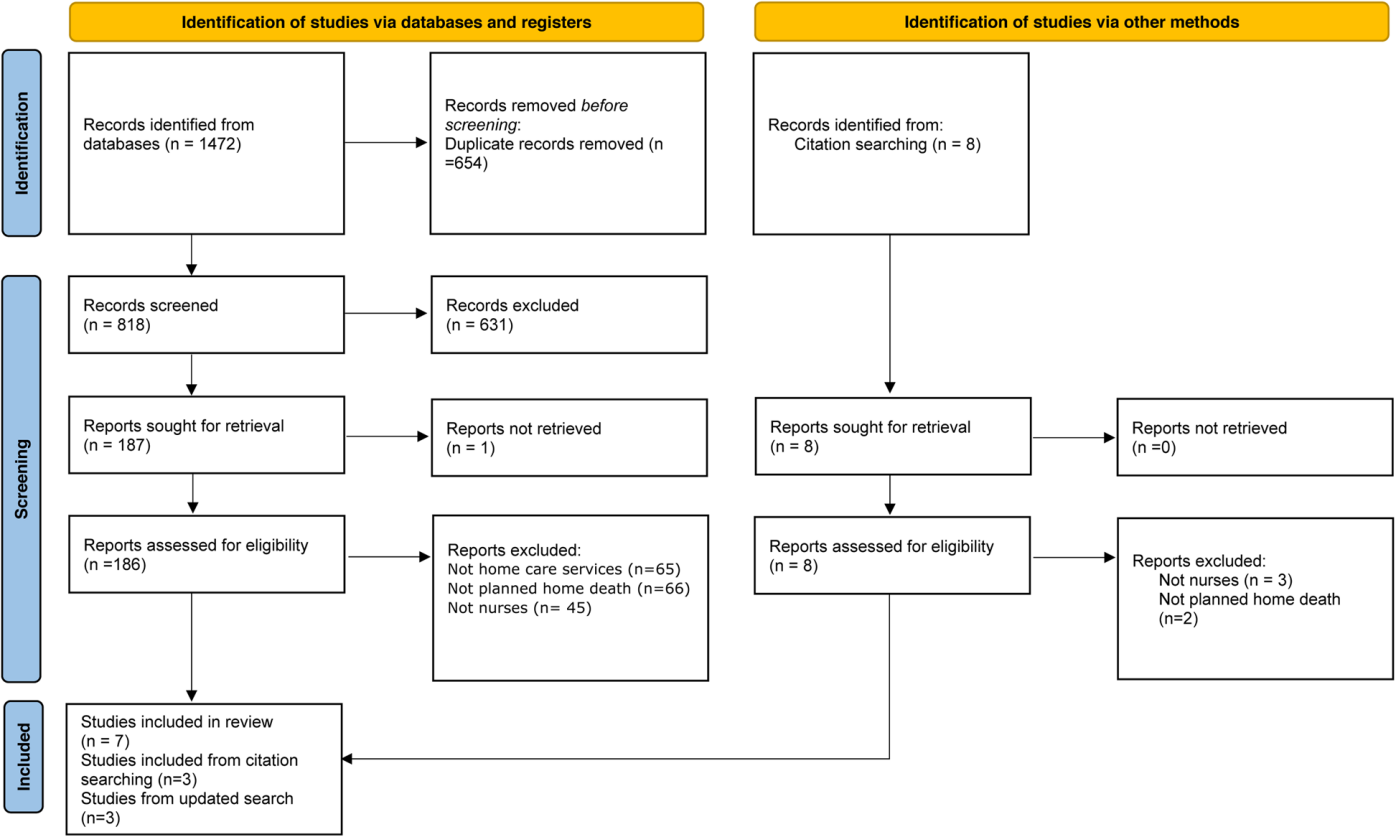
PRISMA flow diagram of search and study selection process.  From: Page MJ, McKenzie JE, Bossuyt PM, Boutron I, Hoffmann TC, Mulrow CD, et al. The PRISMA 2020 statement: an updated guideline for reporting systematic reviews. BMJ 2021;372:n71. doi: 10.1136/bmj.n71

Systematic searches of the electronic databases returned 1,472 records (Fig. 1). Following removal of 654 duplicates, we screened titles and abstracts. 186 full text articles were retrieved. We examined the full text to confirm compliance with our eligibility criteria. Thirteen reports, each representing one study, met the inclusion criteria and made the final data set.

### Characteristics of included studies

All reports were based on studies published as peer-reviewed articles in international journals (Table 1).

**Table 1:** Characteristics of included studies

Author(s)/Year/country	Aim	Study Population	Setting	Methodology and data collection
Burt J, Shipman C, Addington-Hall J, White P. (2008), UK	To explore CNs’ perceptions of their palliative care role, and their provision of such care within the context of their wider generalist workload.	51 community nurses. Not specified male/female	Primary care trusts	Qualitative, focus groups
Cumming, M., Boreland, F., Perkins, D. (2012), Australia	To explore the experiences of community primary health nurses and to determine how personally and professionally equipped they felt for palliative care service provision	34 primary health care nurses. 33 female/1 male.	Rural Palliative care	Qualitative, mailed survey and follow up interviews
Danielsen, B.V. Sand, A.M.; Rosland, J.H.; Forland, O. (2018), Norway	To achieve more insight, through home care nurses and general practitioners, of conditions that facilitate or hamper more time at home and more home deaths for patients with terminal disease and short life expectancy.	7 home care nurses. Not specified male/female.	Palliative care	Qualitative approach with a phenomenological dimension. Focus groups
B. Ervik, B. Brøndbo and M.-L. Johansen (2021), Norway	To explore what healthcare professionals consider necessary to provide equality in care for palliative patients in rural areas	52 Health care professionals, including 30 nurses (15 district nurses, 15 cancer nurses). All female nurses.	Rural palliative care	Qualitative, exploratory, and interpretative approach, with focus group discussions (FGDs) and individual interviews
Ervik, B. Dønnem, T. Johansen, M. L. (2023), Norway	To gain deeper insight into HCPs’ experiences and reflections on home and home death in rural Northern Norway.	52 Health care professionals, including 30 nurses (15 district nurses, 15 cancer nurses). All female nurses.	Rural Palliative care	Qualitative, focus groups and individual interviews. Realist paradigm.
Goodridge, D., and Duggleby, W. (2010), Canada	To explore the opportunities and issues affecting the provision of high-quality palliative care from the perspective of nurses employed in two rural health regions.	44 nurses. 41 female/3 men	Rural Palliative care	Qualitative, focus groups and interviews
Griffiths, J., Ewing, G., and Rogers, M. (2013), England	To present findings of a multi-perspective study that explored how district nurse early support visits are both described and carried out.	58 district nurses. Not specified male/female	Primary care trusts	Qualitative, focus groups and observations and interviews
Jack, B and O`Brien, M. (2010) UK	To explore district nurses and community specialists palliative care nurses’ perceptions and experiences of the factors that influenced hospital admissions of patients with cancer in the final stages of life.	11 district nurses. Not specified male/female	Palliative care	Qualitative method, focus groups
Karlsson, C. and Berggren, I. (2011), Sweden	To describe 10 nurses’ perceptions of significant factors that contribute to good end-of-life care in the patient’s own home	10 Home care nurses. Not specified male/female	End of life care	Qualitative, interviews, Phenomenological hermeneutical method
O’Brien, M. and Jack, B. (2010), England	To explore the views of community nurses (district nurses and specialist palliative care nurses) regarding end-of-life care and the place of death for patients with cancer.	19 district nurses and clinical nurse specialists in palliative care. Not specified male/female	Primary care trusts	Qualitative, focus groups
Smith, R. and Porock, D. (2009),England	To uncover the attitudes of community nurses to the care of the dying patient and the factors that influence these attitudes, including training and education.	158 community nurses. Not specified male/female	Primary care trusts	Quantitative and qualitative survey. Inferential analysis, non-parametric tests and content analysis
Sørstrøm, A.K, Ludvigsen, M.L. and Kymre, I.G. (2023), Norway	To explore home care nurses’ facilitation of planned home death to better understand nursing practices	20 home care nurses. 17 female, 3 men.	Home care service	Qualitative. Roper and Shapira’s qualitative framework on focused ethnography design, Interviews, observations and register forms
Teruya, N., Sunagawa, Y., Sunagawa, H., Toyosato. (2019), Japan	To clarify visiting nurses’ perspectives on critical practices to ensure they could advocate for patients who prefer to die at home.	16 visiting nurses. All female	Homevisit nursing agencies	Qualitative, interviews and observations

The 13 studies published between 2008 and 2023 were conducted in the United Kingdom (*n* = 5) [25–29], Norway (*n* = 4), [30–33], Australia [34], Sweden [35], Canada [36] and Japan [37]. The studies yielded a total number of 443 nurses, ranging from 7 to 158. Among them, 137 were women and 7 were men. Only five out of the 13 studies provided data regarding the gender of the nurses [31–33, 36, 37].

Eleven of the 13 studies were qualitative, and two were quantitative surveys in combination with qualitative interviews [29, 34]. Four studies used focus groups [25, 27, 28, 30], and three combined focus groups with interviews [31, 32, 36]. Two studies combined interviews with observations [33, 37], and one combined focus groups, interviews and observations [26]. One study solely used interviews [35], and one used mailed surveys with follow-up interviews [34].

We identified five issues relating to nurses’ care for patients with home as their preferred place of death: competence, resource limitations, flexibility as a coping mechanism, collaboration, and family caregivers (Table 2).

**Table 2:** Issues of nurses’ care for patients with home as their preferred place of death

Author/ year/	Resource limitations	Competence	Flexibility as a coping mechanism	Family caregivers	Collaboration
Burt, J., Shipman, C., Addington-Hall, J., White, P. (2008) [25]	X	X	X		X
Cumming, M., Boreland, F., and Perkins, D. (2012) [34]		X	X	X	X
Danielsen, B.V., Sand, A.M., Rosland, J.H., Førland, O. (2018) [30]		X	X	X	X
Ervik, B., Brøndbo, B. and Johansen, M.L. (2020) [31]		X	X		X
Ervik, B., Dønnem, T. and Johansen, M.L. (2023) [32]	X		X	X	X
Goodridge, D. and Duggleby, W. (2010) [36]	X	X	X		X
Griffiths, J., Ewing, G., and Rogers, M. (2013) [26]	X	X		X	
Jack, B. and O’Brien, M. (2010) [27]				X	X
Karlsson, C., Berggren, I. (2011) [35]		X		X	X
O’Brien, M. and Jack, B. (2010) [28]	X	X	X		X
Smith, R. and Porock, D. (2009) [29]	X	X		X	X
Sørstrøm, A.K., Ludvigsen, M.S. and Kymre, I.G. (2023) [33]	X	X	X	X	
Teruya, N., Sunagawa, Y., Sunagawa, H., Toyosato, T. (2019) [37]				X	X

### Resource limitations

In 7 out of 13 studies, resource limitations were an issue. Resources can be seen as staffing, items of equipment and nursing supplies. Several studies indicated that a lack of resources serves as a barrier for death at home, and these resources included staff [28–30, 32, 36], time [34] and equipment [28, 33, 35]. Lack of time was correlated with high workloads [33, 34]. Understaffing in combination with long driving distances and high caseloads generated time pressure impediments to nurses’ provision of quality palliative care. Four included articles discussed nursing care for patients dying at home in rural areas. These studies were from countries with scarcely populated areas and long driving distances, such as Norway [31–33], Canada [36] and Australia [34]. In rural areas, the dispersed settlements and long distances pose challenges, particularly at weekends and on holidays when ferries only operate during the daytime, or when weather conditions complicate logistics. This leaves some communities without access to nurses at night. Patients living far from community centres receive fewer home visits, leading to perceptions of unfairness, underprioritised and inconsistent care provision [31, 32]. The time-consuming travel distances strain nursing resources, especially for patients requiring frequent visits or experiencing prolonged end-of-life processes. Nurses often feel isolated without support to discuss their concerns [34]. Efficient use of human resources is challenging, as rural nurses must juggle multiple professional roles, sometimes resulting in role strain. Patients often experience delays in receiving supplies and equipment not readily available in rural settings, highlighting a tension between efficiency and timeliness in rural healthcare [36]. Consequently, promises of death at home are often qualified with “if possible”, emphasising the need to manage expectations [32].

Staffing shortages, as well as difficulties with recruitment and retention of nurses, caused stressful workdays for nurses and as a result a chronic challenge, which in turn caused heavier workloads [25, 33]. Staff shortages had negative consequences for the quality and effectiveness of the care provided, and the lack of adequate home care nursing services was detrimental to patient outcomes, as the nurses provided several examples of patients being prematurely admitted to institutions as a result of not enough nurses to support them in their homes [36], or of patients not being presented with the option of dying at home [33]. The unpredictable nature of the workload was also a complicating factor [25].

### Competence

In 10 out of 13 studies, competence was an issue. Many of the studies referred to the complexity of caring for patients who had home as their preferred place of death. Some nurses spoke of a need for education to better prepare them for care for the dying patient [33], as well as pain and symptom management [36]. Nurses stated that the lack of knowledge jeopardised patient safety, as opioids were not appropriately prescribed or administered [36]. Nurses had been observed to embrace the core principles of palliative care philosophy, emphasising comfort and support without pursuing curative measures [36]. One study suggests that a deficiency in formal training regarding palliative care principles may lead nurses to overlook the fundamental philosophy of palliative care. Consequently, they may persist with invasive procedures even when they are no longer necessary [36].

Ensuring adequate symptom management was also crucial for fostering a sense of safety. Additionally, when nurses demonstrated confidence in their professional competence and technical skills, patients and their families placed trust in them and felt secure about the care provided. However, concerns and uncertainty emerged before nurses visits, as not all nurse possessed the same level of proficiency, leading to preferences among family members for certain nurses over others [35].

Nurses regarded a lack of knowledge as a significant barrier to effective care for patients dying at home [33]. Some of the studies reported a lack of knowledge due to the intermittent nature of palliative care cases, as there would be long periods of time between each patient who wanted to die at home [33], which affected the sense of clinical competence [33, 34]. Nurses stated how they wanted more experience in caring for patients wanting to die at home, in order to get training and practice [33].

Many nurses did not find training to care for the dying available or easy to access [29]. Barriers to formal education were stated as competing work roles, work load, geographical distances and lack of backfill [34]. They found digital education useful, but they often found themselves unable to make time to join, due to work load pressures [34]. Access to written guidelines and practical tools was deemed crucial when providing nursing care for patients who had home as their preferred place of death [31], yet maintaining evidence-based practice for nurses who provide a wide range of services in addition to palliative care in the patients home was challenging [34]. A few nurses had undertaken a Program of Experience in the Palliative Approach (PEPA), involving a 3–5 day supervised placement at a specialist palliative care service, which was reported as useful [34]. Nurses emphasised that insufficient education further hindered staff’s capacity to deliver effective palliative care. They highlighted the primary concern as the shortage of appropriately skilled staff within care agencies, which could endanger the well-being of certain patients [28, 36].

### Flexibility as a coping mechanism

Study participants from 5 included articles valued being able to care for people with home as their preferred place of death. Nurses considered it rewarding, or synonyms thereof [25, 29, 32, 33, 36]. Nurses reported a high degree of satisfaction from caring for the dying and achieving a ‘good death’ [29]. High levels of commitment to these patients were a motivator for nurses when providing care in challenging conditions [25, 31–33].

However, nurses mitigated the consequences of resource constraints on the access and quality of care by serving out of hours, skipping breaks, working overtime, or going “above and beyond” [31, 33]. Some nurses explained that they wanted extra time on the first days to establish trust and a relationship with the patients and next of kin, and that they had the ambition to send an experienced nurse to the first meeting. However, this was not always an option as they had limited resources and many patients [30].

Studies describe the care provided for palliative patients as “above and beyond”, “out of their way”, doing more than expected and prioritising these patients, such as with visits at specific times, making sure that key staff were available by altering shift patterns, and making patient visits on days off. Putting a folding cot in the office and sleeping in a sleeping bag in the patient’s home are examples of commitment and displays of flexibility [33, 34]. From one study with a rural focus, human healthcare resources within the local community were utilised flexibly. Patients dying at home occasionally stayed at the nearby nursing home for a few days, or nurses from nursing homes provided care directly in the patients’ homes. In areas with limited nursing staff or without available nurses on at nights, a system of continuous 12-hour shifts was sometimes implemented, to ensure medication administration, with nurses relieving each other as needed [31]. Nurses slot palliative patients in wherever possible in their schedule, ensuring that both patient and NOK receive prioritised attention, availability, and flexibility [25, 33]. They refrain from recording time spent during visits, and give their phone number for the patients or NOK to use if needed [30, 33] Some recounted instances where they rearranged or cancelled their planned holidays to ensure nurse coverage, especially during a summer when a patient in their home care area wished to spend their final days at home [33].

### Family caregivers

Collaboration with the families was highlighted in 9 out of 13 articles. Family caregivers were seen as a major factor for being able to provide care for patients with a preference to die at home, and it is almost unfeasible to achieve death at home without [27, 30, 32, 33]. A key element contributing to safety was the presence of supportive family caregivers who were both willing and brave enough to stay with the dying patient [35]. In rural settings, nurses generally regarded it as advantageous when they had prior knowledge of patients and had established longstanding relationships with them before they required palliative care [36]. Nurses dedicated significant time and effort to support and educate family caregivers, ensuring they felt confident in their caregiving role and could recognise changes in the patient’s condition [26]. Despite this collaboration, some caregivers did not grasp the patient’s terminal status and were disappointed by the limitations of healthcare professionals in home care. For some, the situation became overwhelming and frightening, prompting them to seek assistance by discussing the patient’s condition with nurses. Often, these discussions led to the patient’s transfer to a local nursing home or palliative unit [32].

Barriers to achieving death at home were evident from the heavy burden placed on caregivers, who could become too exhausted to feel safe providing care at home, despite the patient’s wishes [33]. Nurses provided essential support, offering round-the-clock consultation and symptom management, and empowering families in their caregiving [37]. Participants expressed pride in the patient-centredness of palliative care, emphasising the value of knowing patients beforehand and fostering long-standing relationships, which enhanced person-centred care and outcomes [36]. Safety in caregiving was found in the presence of confident family members willing to stay with the dying person, creating a sense of security and support [35]. The capacity of family caregivers to manage their responsibilities significantly influences adherence to a patient’s preference concerning the place of death. However, family caregivers frequently reach a threshold where they are no longer able to cope, often occurring in the advanced stages of the illness. Tensions can arise when they feel incapable of fulfilling the patient’s wish to pass away at home, even with support available from healthcare services. Patients may contemplate altering their preferred place of death out of a sense of concern for others, rather than prioritising their own preferences and needs [27].

None of the studies included described specific instruments used in the support of home as the preferred place of death, although assessments were conducted of patients and caregivers, with emphasis on physical well-being. Visits involved evaluating physical symptoms like pain, breathlessness, appetite loss, nausea, and fatigue, alongside medication effectiveness. Activities of daily living such as mobility, eating, sleeping, self-care and continence were regularly assessed [26]. Caregivers’ concerns were addressed, including patients’ appetite changes, smoking, pain management, and caregivers’ own physical needs and ability to provide care. Additionally, the need for mobility aids like wheelchairs and TENS machines was considered [26]. However, the assessments relied solely on conversational methods without formal evaluations. Other studies examined the potential to extend home care until the patient’s passing, considering factors like caregiver burden, the patient’s condition, and family readiness, without using formal assessment forms [33, 37].

### Collaboration

In 11 of the 13 articles, collaboration with other healthcare professionals was highlighted as important for nursing care for patients with home as their preferred place of death. Collaboration included general practitioners (GPs) [29, 35, 37], the referring hospital [28, 30], nursing homes [31] and palliative teams/multidisciplinary ambulatory teams [29, 30]. Some had agreements on how to efficiently collaborate with them, such as electronic messaging between GPs and nurses [30].

However, collaborations were also challenging. Early and unprepared discharge from hospital was mentioned in two studies [27, 30], and nurses expressed frustration when patients with a short life expectancy and an expressed wish to die at home, were discharged on Fridays [28, 30]. Insufficient information provided to the nurses was compounded by a lack of detailed guidance regarding patient needs upon discharge from hospital. Occasionally, what initially seems to be a routine visit for nurses turns out to be a far more intricate and demanding case [28].

Hospital staff may lack a comprehensive understanding of the responsibilities and capabilities of nurses, potentially resulting in the promise of services that are not feasible [27, 30]. This in turn can lead to nurses having to disappoint and explain to patients and their families which services were within their capacity. Some nurses also saw that the families had a harder time at home without the “*safety net*” of the hospital, causing carer breakdown [27]. Since the original plan for dying at home could become challenging, having an alternative plan was deemed crucial. Ideally, discussing the option of transitioning to a nursing home or hospital early on was preferred, to prevent overwhelming circumstances. Such transitions were sometimes prompted by staffing shortages, but often stemmed from multifaceted issues such emergencies, infections, and uncontrolled symptoms [32]. Nurses identified the absence of coordination with community teams and the lack of appropriately planned and organised care packages as crucial barriers to dying at home [28].

Caring for a significant number of palliative care patients in their own homes can impose considerable stress and strain on nurses, who often lack formal support services to help them process their emotions regarding palliative care provision and other job-related pressures [25, 30]. For some nurses, their teams and neighbouring teams served as the primary source of informal support, offering both practical assistance and emotional comfort during challenging times. There was a prevalent sentiment in one study that formal support would be greatly beneficial [25].

## Discussion

Through a scoping review incorporating 13 primary studies, we systematically examined and systematized the existing evidence pertaining to nurse’s care for patients who have home as their preferred place of death. Nurses, particularly in rural settings, need to prioritise embracing palliative care principles that prioritise comfort over aggressive treatments. This emphasis may sometimes be overlooked, due to insufficient formal training, but it is crucial for addressing the unique challenges faced in rural palliative care, including dispersed populations and long distances that impact timely access to healthcare services and resources [38]. Furthermore, human resource management and logistical issues present additional challenges in rural palliative care delivery. Assessment tools focusing on various aspects of patient well-being are underutilised, highlighting potential gaps in care provision. However, the use of electronic communication tools shows promise in improving collaboration among healthcare providers, potentially addressing some of the logistical challenges.

Positive outcomes in palliative care are often associated with the ability to facilitate planned home deaths based on patient preferences. The involvement and dedication of nurses are crucial for achieving such outcomes. Nonetheless, understanding the differences in experiences and outcomes, depending on where individuals die, is essential [39]. While some evidence suggests that psychological, social and holistic measures of patient well-being may be better addressed at home, findings concerning symptoms and family outcomes lack consistency, making it challenging to definitively compare dying at home to dying in hospital [39].

The principal findings encompass competence, resource limitations, flexibility as a coping strategy, collaboration, and family caregivers. The care for patients that has home as their preferred place of death reveals conspicuous challenges in service delivery. These challenges stem from the intricacy of end-of-life patient care, compounded by the context of delivering care within patients’ residences.

The shortage of appropriately skilled staff within care agencies is a significant concern that directly impacts patient well-being. Insufficient time, supplies and staff exacerbate stress levels, posing risks at both organisational and personal levels. Similar findings have been supported by prior research [40, 41]. Additionally, the absence of necessary equipment limits the capacity of nurses to deliver essential care, potentially leading to feelings of embarrassment among nurses when interacting with other healthcare providers [42]. Moreover, it is essential to recognise that resources extend beyond staffing, equipment and nursing supplies; education also plays a crucial role [15]. Nurses advocate for more training opportunities and practice in handling death at home, particularly in areas such as palliative care and pain management. Deficiencies in knowledge have been identified as compromising patient safety, highlighting the need for enhanced education in these areas.

Furthermore, the intermittent nature of cases where patients have home as their preferred place of death exacerbates the lack of knowledge as a significant barrier to effective care [43]. These findings underscore the importance of addressing nurses’ educational needs to ensure the delivery of high-quality care for patients who wants to die at home. Lack of knowledge emerges as a substantial barrier to effective care. Access to formal education is hindered by various factors such as competing work roles, workload, geographical distances, and lack of backfill. While digital education offers some benefits, time constraints remain a hindrance. Despite recognition of education limitations and barriers to enhancing competence in previous studies [43–45], our findings suggest that these issues persist. However, nurses demonstrate remarkable dedication to prioritising patient well-being. They frequently reorganise their schedules and make personal sacrifices to ensure continuous coverage for palliative patients dying at home. Nurses undertake case management responsibilities, facing challenges in reconciling client-centred and system-centred objectives. They play a vital role in coordinating care among interconnected parties, navigating challenges with information sharing and communication systems [46]. Nevertheless, poor inter-professional communication and uncoordinated discharge processes contribute to patients’ final wish to die at home not being fulfilled. This can result in delayed access to services and ultimately lead to patients passing away in hospital settings [47]. Addressing these communication challenges and ensuring coordinated discharge planning are essential for improving the quality of end-of-life care effectively.

Findings from the current review highlight the crucial role of family caregivers, suggesting that to die at home may be inconceivable without their involvement. Collaboration between nurses and caregivers is emphasised, with nurses actively engaging in motivating, guiding, and supporting caregivers. Supportive family has been shown to be an enabling factor associated with home death [41]. However, despite the pivotal role of caregivers, no formal assessments to evaluate their capacity were described in this review. Nurses also express concern about the burden placed on caregivers. Caregivers may become overwhelmed to the extent that they no longer feel capable of caring for patients at home, despite the patient’s desire to remain there. This indicates that caregivers have unmet needs that nurses recognise but are unable to fully address. Similar findings suggest that nurses perceive and acknowledge caregivers’ needs for emotional support, guidance, and decision-making assistance. However, these assessments often rely on intuition and experience, rather than a systematic approach [48]. Overall, these findings underscore the importance of recognising and addressing the needs of family caregivers when patients want to die at home. Implementing formal assessments and providing targeted support may help alleviate the caregiver burden and ensure the well-being of both caregivers and patients during end-of-life care.

### Limitations

The lack of information on demographic factors such as gender in several of the studies included is a limitation. The inclusion criteria focused specifically on studies related to nurse’s care for adult patients with home as the preferred place of death. Exclusion of studies involving other healthcare professionals or different age groups may limit the generalisability of the findings to broader healthcare contexts or different populations. Only including studies published in the English language may introduce publication bias, as relevant studies published in other languages could be overlooked. This limitation may result in an incomplete representation of the available evidence, potentially biasing the findings of the review. The exclusion of conference papers may result in the omission of valuable insights and preliminary findings presented in conference settings, as conference papers often provide timely information on emerging research trends and findings, and their exclusion may limit the comprehensiveness of the review’s findings.

## Conclusions

In conclusion, the examination of nursing care for patients with home as the preferred place of deaths through a scoping review of 13 primary studies has underscored challenges in service delivery. These included shortages of skilled staff, inadequate resources, and deficiencies in education. Despite recognising these challenges, issues such as a lack of knowledge and barriers to enhancing competence persist. Nonetheless, nurses demonstrate remarkable dedication to prioritising patient well-being, often making personal sacrifices to ensure continuous coverage, and address complex patient needs. Collaboration with family caregivers is crucial in enabling death at home, although concerns about caregiver burden and unmet needs continue. Moving forward, addressing these challenges, and enhancing support for nurses and family caregivers, will be essential when caring for patients who want to die at home.

## Supplementary Information

Supplementary Material 1.

Supplementary Material 2.

## Data Availability

No datasets were generated or analysed during the current study.
