# Supplementary material for: Nursing care to patients who have the home as the preferred place of death: a scoping review
Source: BMC Health Serv Res. 2024 Oct 29;24:1302. doi: 10.1186/s12913-024-11757-8 (PMC11520454; doi:10.1186/s12913-024-11757-8)
Supplement: Supplementary file 1 — Supplementary Material 1. [file 12913_2024_11757_MOESM1_ESM.docx]

**Database:**
Ovid MEDLINE(R) <1946 to January Week 2 2024>

| **#** | **Query** | **Results from 19 Jan 2024** |
| --- | --- | --- |
| 1 | Home Care Services/ | 36,631 |
| 2 | home care service?.tw,kf. | 2,436 |
| 3 | home health care.tw,kf. | 2,838 |
| 4 | Nurses, Community Health/ | 1,019 |
| 5 | Community health nurse?.tw,kf. | 731 |
| 6 | 1 or 2 or 3 or 4 or 5 | 39,829 |
| 7 | home death?.tw,kf. | 547 |
| 8 | dying at home.tw,kf. | 486 |
| 9 | death? at home.tw,kf. | 372 |
| 10 | die at home.tw,kf. | 723 |
| 11 | 7 or 8 or 9 or 10 | 1,713 |
| 12 | 6 and 11 | 636 |
| 13 | Terminal Care/ | 32,174 |
| 14 | terminal care.tw,kf. | 2,508 |
| 15 | Palliative Care/ | 64,047 |
| 16 | palliative care.tw,kf. | 35,598 |
| 17 | end-of-life care.tw,kf. | 12,588 |
| 18 | end-of-life nursing.tw,kf. | 146 |
| 19 | palliative nursing.tw,kf. | 282 |
| 20 | 13 or 14 or 15 or 16 or 17 or 18 or 19 | 97,422 |
| 21 | 6 and 11 and 20 | 552 |
| 22 | limit 21 to English language | 480 |
