# Supplementary material for: Nursing care to patients who have the home as the preferred place of death: a scoping review
Source: BMC Health Serv Res. 2024 Oct 29;24:1302. doi: 10.1186/s12913-024-11757-8 (PMC11520454; doi:10.1186/s12913-024-11757-8)
Supplement: Supplementary file 2 — Supplementary Material 2. [file 12913_2024_11757_MOESM2_ESM.docx]

Appendix II: Studies excluded on full text

*Example:*

Lai, X.B., Chen, L.Q., Chen, S.H. and Xia, H.O. (2019) An examination of home-based end-of-life care for cancer patients: a qualitative study

*Reason for exclusion:* Not home care service
